# Editorial: Hormones and receptors in breast cancer

**DOI:** 10.3389/fendo.2023.1185615

**Published:** 2023-03-28

**Authors:** Cinthia Rosemblit, Norbert Nass, María Celeste Díaz Flaqué

**Affiliations:** ^1^ National Scientific and Technical Research Council (CONICET) Institute for Biomedical Research (BIOMED), Buenos Aires, Argentina; ^2^ Städtische Klinikum Dessau, Dessau-Roßlau, Germany

**Keywords:** breast cancer, hormones, receptors, biomarker, cancer etiology

Breast cancer (BC) is the most common type of cancer and the fourth cause of cancer death among women worldwide. Steroid (estrogens, progestogens, androgens and glucocorticoid), thyroid, TSH, prolactin and growth/insulin receptors signaling pathways activated by their hormones have a major role in the mammary gland development and in the etiology, and progression of BC. Although classification of BC is defined by hormone receptor expression, (estrogen receptor-ER and/or progesterone receptor-PR), there is evidence indicating that estradiol and progesterone are not the unique hormones driving BC progression.

During the last years, the role of different hormones has been unravelled in biological aspects to regulate tumor progression, metastasis, and treatment response, as well as their receptors crosstalks. The study of hormonal receptors expression as well as the molecular mechanism induced could have a prognostic value in BC.

The present Research Topic aims to provide a platform for novel research in BC, including original research articles that would help in the development of better diagnostic, prognostic and/or therapeutic strategies for this disease.

ER remains the most important biomarker in breast oncology. ER^low^ tumors represent a relatively small subgroup of BC patients, being similar to ER^neg^ disease in their molecular landscape, clinicopathological characteristics, and response to therapy. Nevertheless, a proportion may retain some ER signalling dependency, and so the possibility of responding to some degree to endocrine therapy and should be considered as a heterogeneous number of tumors to be fully characterized from the molecular point of view. Reinert et al. reviewed the most important considerations regarding the definition of ER positivity, pathology assessment, prognosis, and therapeutic implication of ER^low^ BC from the medical oncology perspective. Whether to offer endocrine therapy as part of the overall strategy under the possibility of some remaining endocrine sensitivity should remain an individual discussion.

Wright et al. analysed the signalling network of PR in a BC model cell line, T47D by phospho-proteomics. Similar to ER, PR signals mostly through the genomic pathway, but membrane bound forms have also been described. The T47D cell line is a model often applied for PR research; however, the phospho-proteome determination combined with pathway enrichment analysis provides new insights into the molecular function of this hormone. Especially, these data suggest progesterone mediated changes in the nuclear structure and epidermal mesenchymal transition (EMT). Interestingly, neurotrophin and insulin signalling seems also activated. Further studies should show whether these predictions originating from a single cell line hold true for clinical BC.

The androgene receptor (AR) is not a classical prognostic factor in BC, however, it is expressed in many BC and also characteristic for one subtype of triple negative BC. Several drugs are available and applied clinically for prostate cancer, so it is tempting to propose AR as target for BC as well. In this Research Topic, Ravaioli et al. review our current knowledge on this receptor and aim at answering important questions for evaluating the role of AR in BC. This means defining the structure and function of this protein, and how to detect this receptor diagnostically and in which subtype or stage it might be important. Altogether for AR-positive triple negative cases, new therapies targeting AR might be evolving.

In the same line, Chen et al. analysed the published papers to find which journals fancy AR research, the most active research institutions, influential researchers, the most important research papers and keywords worth to further explore, providing guidance for researchers who are interested in this field. Endocrine therapy is milder and more acceptable to patients than chemotherapy. Thus, if a new kind of drugs that have completely different mechanisms from existing endocrine therapy is available, it is possible to prolong patients’ lifetime. With the help of this scientometric analysis in this field, researchers can clarify the current research status and hot spots worth to fully explore.

Related to the finding that insulin signalling seems activated by progesterone, Dittmer reviews our current knowledge on the insulin-like growth factor-1 (IGF-1) binding proteins, especially IGFBP5. It is well known that IGF-1 signalling is associated with a major clinical problem, the acquired resistance to the selective ER modulator tamoxifen or the ER degrader fulvestrant. Here, IGFBP5 seems to be a major player by modulating signalling *via* BCL3 or the ER, but also affecting ERK signalling. Interestingly, the extracellular IGFBP5 can inhibit or promote IGF-1 signalling, depending on the cellular context such as the tumor micro environment. Dittmer points out that IGFBP5 can also have IGF-1 independent effects, by binding to a membrane receptor, leading to internalization and accumulation in the nucleus. IGFBP5 might therefore become an important prognostic biomarker or drug target in the future.

In a case report, Liu et al. describe a rare, hypoglycemic coma due to a borderline phyllodes tumor of the breast. The most common cause of non-islet cell hypoglycemia of this type is tumoral overproduction of incompletely processed IGF-2, which stimulates insulin receptors and increases glucose utilization. The patient’s hypoglycemia resolved rapidly after the removal of the breast tumor. Pathological examination confirmed a borderline phyllodes tumor of the breast, and immunohistochemical staining showed high expression of IGF2 in the tumor tissue. According to research, these patients frequently have severe hypoglycemia, impaired consciousness, a giant breast tumor, and detectable big-IGF2 in serum or tumor tissue.

Pathological complete response (pCR) is considered a surrogate for favorable survival in BC patients treated with neoadjuvant chemotherapy (NACT). The study by Qian et al. aimed to develop and validate a nomogram for predicting the pCR probability of BC patients after NACT based on the clinicopathological features. A nomogram is considered to be a reliable tool to predict the prognosis of cancer patients. This nomogram is based on age, AJCC T stage, Ki67 index before NACT, HER2, and HR status, which can be non-invasively applied to personalize the prediction of pCR in BC patients treated with NACT both before NACT and before surgery. The nomogram has the potential to assist clinicians in screening BC patients for NACT and adjusting the optimal surgical approach for BC patients after NACT.

Shi et al. constructed another nomogram to predict axillary pCR after neoadjuvant systemic therapy (NST) in clinically node-positive patients with BC. Efficacy indicators reflecting the therapeutic response of NST were found to be very important in predicting axillary pCR. Based on these efficacy indicators and some baseline indicators, they established a novel nomogram, which was validated and considered to be highly accurate in predicting axillary pCR. This predictive model may help surgeons to de-escalate or even omit axillary surgery for patients with axillary lymph node downstaging after NST in the future.

Overall, the articles outlined in the current Research Topic introduce new relationships between biological factors and BC markers, diagnostic, and/or prognostic approaches for better management of this disease.

## Author contributions

All authors listed have made a substantial, direct, and intellectual contribution to the work and approved it for publication.

